# Supplementary material for: Identification of photoperiod-induced specific miRNAs in the adrenal glands of Sunite sheep (Ovis aries)
Source: Front Vet Sci. 2022 Jul 22;9:888207. doi: 10.3389/fvets.2022.888207 (PMC9354845; doi:10.3389/fvets.2022.888207)
Supplement: Supplementary file 1 [file Data_Sheet_1.ZIP › Supplementary file/miRDeep2 file.zip!/miRDeep2 file/Know_Structure/SPLP42A1/oar-mir-411a.pdf]

miRBase precursor : oar-mir-411a
Total read count : 42033
oar-miR-411a-5p read count : 38974
oar-miR-411a-3p read count : 3054
remaining reads : 5

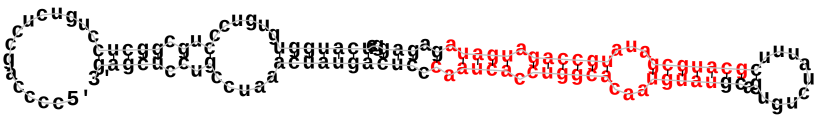

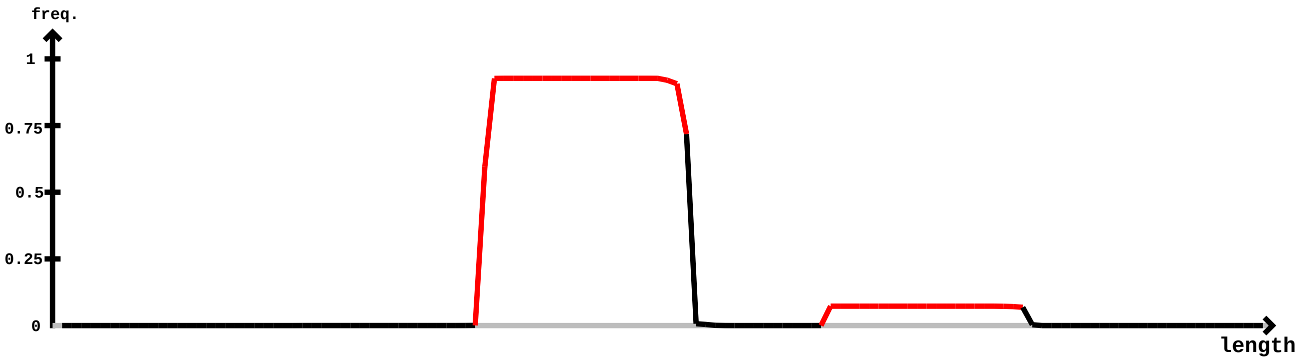

oar-miR-411a-3p

oar-miR-411a-5p

```
5'- ccccagccucuguccucggcgucccugugugguacucggagagauaguagaccguauagcguacgcuuuaucugugacguauguaacacgguccacuaacccucaguaucaaauccguccucgag  -3'   exp
    ..............(((((.(.(......(((((((..(((.(.((((.((((((...((((((((.........).)))))))...)))))).)))).).))))))))))).....).).)))))   reads    mm
    ......................cccugugugguacucggag....................................................................................   1        0
    .......................ccugugugguacucggagag..................................................................................   1        0
    ........................cugugugguacucggagaga.................................................................................   2        0
    .........................................agauaguagaccguauagcgu...............................................................   1        0
    .........................................agauaguagaccguauagcgua..............................................................   1        0
    .........................................agauaguagaccguauagcguac.............................................................   2        0
    ..........................................gauaguagaccguauagcgu...............................................................   4        0
    ..........................................gauaguagaccguauagcgua..............................................................   1        0
    ..........................................gauaguagaccguauagcguac.............................................................   2        0
    ..........................................gauaguagaccguauagcguacg............................................................   10       0
    ...........................................auaguagaccguauagc.................................................................   3        0
    ...........................................auaguagaccguauagcg................................................................   8        0
    ...........................................auaguagaccguauagcgu...............................................................   255      0
    ...........................................auaguagaccguauagcgua..............................................................   390      0
    ...........................................auaguagaccguauagcguac.............................................................   4981     0
    ...........................................auaguagaccguauagcguacg............................................................   19253    0
    ...........................................auaguagaccguauagcguacgc...........................................................   60       0
    ...........................................auaguagaccguauagcguacgcu..........................................................   40       0
    ...........................................auaguagaccguauagcguacgcuu.........................................................   2        0
    ............................................uaguagaccguauagcg................................................................   1        0
    ............................................uaguagaccguauagcgu...............................................................   37       0
    ............................................uaguagaccguauagcgua..............................................................   163      0
    ............................................uaguagaccguauagcguac.............................................................   2949     0
    ............................................uaguagaccguauagcguacg............................................................   10607    0
    ............................................uaguagaccguauagcguacgc...........................................................   54       0
    ............................................uaguagaccguauagcguacgcu..........................................................   104      0
    ............................................uaguagaccguauagcguacgcuu.........................................................   23       0
    ............................................uaguagaccguauagcguacgcuuu........................................................   5        0
    .............................................aguagaccguauagcguac.............................................................   2        0
    .............................................aguagaccguauagcguacg............................................................   12       0
    .............................................aguagaccguauagcguacgcu..........................................................   2        0
    .............................................aguagaccguauagcguacgcuu.........................................................   1        0
    ..............................................guagaccguauagcguacg............................................................   1        0
    .......................................................auagcguacgcuuuaucugugacguauguaacacggucc...............................   1        0
```

```
                                                                                oar-miR-411a-3p
                                           oar-miR-411a-5p
ccccagccucuguccucggcgucccugugugguacucggagagauaguagaccguauagcguacgcuuuaucugugacguauguaacacgguccacuaacccucaguaucaaauccguccucgag

..............................................................................guauguaacacgguccacuaa..........................    1    0
..............................................................................guauguaacacgguccacuaac.........................    2    0
...............................................................................uauguaacacgguccacu............................    19   0
...............................................................................uauguaacacgguccacua...........................    41   0
...............................................................................uauguaacacgguccacuaa..........................    114  0
...............................................................................uauguaacacgguccacuaac.........................    2746 0
...............................................................................uauguaacacgguccacuaacc........................    130  0
................................................................................auguaacacgguccacuaacc........................    1    0
```
